# Allometric Equations for Aboveground and Belowground Biomass Estimations in an Evergreen Forest in Vietnam

**DOI:** 10.1371/journal.pone.0156827

**Published:** 2016-06-16

**Authors:** Vu Thanh Nam, Marijke van Kuijk, Niels P. R. Anten

**Affiliations:** 1 Department of Biology, Utrecht University, Utrecht, the Netherlands; 2 Vietnam Administration of Forestry, Hanoi, Vietnam; 3 Centre for Crop Systems Analysis, Wageningen University, Wageningen, the Netherlands; Wuhan Botanical Garden,CAS, CHINA

## Abstract

Allometric regression models are widely used to estimate tropical forest biomass, but balancing model accuracy with efficiency of implementation remains a major challenge. In addition, while numerous models exist for aboveground mass, very few exist for roots. We developed allometric equations for aboveground biomass (AGB) and root biomass (RB) based on 300 (of 45 species) and 40 (of 25 species) sample trees respectively, in an evergreen forest in Vietnam. The biomass estimations from these local models were compared to regional and pan-tropical models. For AGB we also compared local models that distinguish functional types to an aggregated model, to assess the degree of specificity needed in local models. Besides diameter at breast height (DBH) and tree height (H), wood density (WD) was found to be an important parameter in AGB models. Existing pan-tropical models resulted in up to 27% higher estimates of AGB, and overestimated RB by nearly 150%, indicating the greater accuracy of local models at the plot level. Our functional group aggregated local model which combined data for all species, was as accurate in estimating AGB as functional type specific models, indicating that a local aggregated model is the best choice for predicting plot level AGB in tropical forests. Finally our study presents the first allometric biomass models for aboveground and root biomass in forests in Vietnam.

## Introduction

Allometric regression models are widely used for estimating tree biomass in forests. These models are mathematical functions that relate tree dry mass to one or more tree dimensions, such as diameter (DBH), height (H) and wood density (WD) [1,2]. A major challenge lies in developing models that are both accurate and relatively easy to use. It has been argued that models based on large compiled data sets (see Brown [1] and Chave et al. [3]) generally perform better for larger scale assessments than local models because the latter are fitted on a limited number of trees [3–5]. However, results from other studies suggest local models to be more accurate on smaller scales [2,6–9].

Most models currently in use are multi-species in the sense that a single allometric equation is developed for all species considered in one or several specific locations. Evidently this ignores the enormous species diversity and associated inter-specific trait variation that exists in tropical forests [10,11]. The use of aggregated models assumes that concomitant tree-level errors in biomass estimates will cancel out at the plot level [9], and the development of species-specific models may not be feasible simply because a sufficient number of sample trees will likely not be available for every species. An alternative is to categorize species by wood density (WD) classes and develop models for each wood density class. WD is believed to be a key trait indicating the ecological strategy of a species, with low WD being associated with high mass-growth rates [12–15] and high wood density with resistance to damage and disease, and shade tolerance. Growth traits of trees are therefore believed to be more similar within than across WD classes (hereafter denoted as functional type) [3]. Furthermore, WD is closely correlated with timber quality traits and forest managers tend to categorize species by WD, for instance the Vietnamese forestry service uses different WD classes to categorize trees. Yet, as far as we know, the use of functional-type specific allometric models has not been considered in tropical forest biomass assessment studies.

Most estimates of tropical forest biomass focus only on aboveground biomass [1,3,5–7,16]. Root biomass (RB) is often estimated as a fraction of the aboveground biomass (i.e., the root-shoot ratio (RS)), with the IPCC [17] recommending a RS value of 0.24 to be used for all tropical moist, dry and secondary forests, respectively, based on Cairns et al. [18]. However, RS values can vary substantially between trees depending on species and growth conditions [19]. A review by Brown [1] found RS values in lowland moist tropical forest to exhibit an 8-fold variation ranging from 0.04 to 0.33 (the mean being 0.12). There is thus an urgent need to use allometric models that can accurately estimate root biomass similar to those used for aboveground biomass, but very few such models currently exist [2,8,20].

While several allometric models for both above and below ground biomass have been developed recently for South East Asian tropical secondary and *Dipterocarp* forests, e.g. Ketterings et al. [16], Basuki et al. [6], Kenzo et al. [7], Kenzo et al. [8], Niiyama et al. [20], no such models exist for Vietnam. Considering the involvement of Vietnam in REDD+ programmes, it is important to develop local models and to assess the degree of specificity that such models should have with respect to locality and species, or functional type specificity.

In this study the following issues are addressed: (i) the difference in biomass estimations between local, and regional and pan-tropical models (ii) the necessity to develop functional type specific models and (iii) the development of an allometric model for root biomass and testing this against existing models (i,e. IPCC model and foreign models).

## Materials and Methods

(The field activities were carried out in the production forests that are managed by the Highland Tropical Forest Research Centre and Kanak Forestry Company, K’Bang District, Gia Lai Province. All of the field activities were permitted by the directors of the companies).

### 2.1 Study site

The study was conducted in an evergreen forest (108° 17’ 75” E and 14° 35’35” N) in K’Bang district, Gia Lai province, in the central highland zone in Vietnam. The topography of the area is mostly flat with an altitude ranging from 500–600 m above sea level. Annual precipitation is approximately 2,300 mm with a 3 to 4-months dry season. Mean annual air humidity is 82% and mean annual temperature is 23°C. The soils in the area are classified as Ferrasols [21]. A map of the location of the study site is provided in the supplementary material (see S1 Fig).

The forest at the study site was selectively logged for the first time between 1980–1982 with a harvesting intensity of about 30–35% of the standing volume and focussing solely on species producing timber suitable for construction. A total of six permanent plots (100 x 100 m each) were established in the study site in 2004 by the Highland Tropical Forest Research Centre (hereafter Highland FRC). The forest was never logged again, therefore the plots had a 30–32 year recovery period during the time of measurements in 2012.

Forest inventory data were collected in the permanent plots between December 2011 and April 2012. In each permanent plot, all trees with a diameter at breast height (DBH) larger than 10 cm were identified [22] and numbered. For each tree, height (H) (using a Blumleiss altimeter) and DBH (with a diameter tape) was measured. In total 105 species were found within these plots.

### 2.2 Measurements of aboveground biomass

In order to parameterize local allometric models, a total of 300 trees pertaining to 45 species were sampled destructively. These trees were sampled in two logging compartments close to our study area during a logging event (Apr—Jun 2012). Sample trees were selected in such a way that their size range (height and DBH) was as much as possible representative of the trees measured in the permanent plots. Information on sample trees can be found in the supplementary file (S1 and S2 Tables).

After felling, diameter (DBH) and height (H) (equal to the length of the stem) of each individual sample tree were measured. In addition, for larger trees (DBH>40 cm), we applied the Smalian’s formula with an interval of two metres [6] to determine the volume of concomitant segments of the stem and big branches. Fresh weight of stems, branches (for trees with a DBH<40 cm) and leaves were determined separately using a balance with an accuracy of ±0.1 kg after which subsamples were taken (see below). We also measured the height and diameter at the cutting surface of each stump that remained after felling, in order to calculate its volume. Dry mass of small trees (DBH<40 cm) and leaves were determined based on the dry weight to fresh weight ratio (DW/FW, see below). For large trees we calculated dry mass by multiplying stem and branch volumes by wood density (see 2.4 for WD determination). For the tree’s stump, the dry mass was determined by multiplying its volume by wood density (see 2.4).

Two wood samples were taken from the lower (at cutting position) and upper parts (under the first branch position) of the stem (maximum 3 individuals per species) with a fresh weight of around 50 g. Each wood sample was stored in a plastic bag to avoid water loss. Wood samples were sent to the Wood Science Laboratory of the Forestry University of Vietnam where fresh mass was determined and where they were oven-dried at 105°C to constant weight. The DW/FW ratio of each wood sample was calculated by the relation between dried weight (DW) and fresh weight (FW). The DW/FW ratio of leaves were determined in a similar way but using subsamples of about 30 g and oven-drying at 65°C.

### 2.3 Measurements of root biomass

Due to the limitation of budget and time, root biomass could not be collected and measured for all the above mentioned sample trees. We limited ourselves to those individuals that had been uprooted by bulldozers in the logging compartments. We were able to determine root mass for 40 of the 300 individual sample trees (25 species) that had been used for the AGB measurements. Roots remaining in the soil after the tree had been uprooted were dug out as best as possible, though we were unable to remove all finer roots (see also Discussion). Roots were divided into smaller parts by a chainsaw and the soil was carefully removed by brushing. The fresh weight of the root system of each individual was determined and a subsample was taken and oven-dried in the Wood Science Laboratory at 90°C to constant weight. Total root mass was calculated by multiplying the total fresh weight of the root system by DW/FW ratio.

### 2.4 Wood density measurement

To determine wood density (WD) for each species, two wood core samples with a length of around 15 cm and a diameter of 0.5 cm were taken from opposite positions on the stem at DBH of the same individuals that were used to determine DW/FW wood ratios. For the five most commonly harvested species (i.e. containing 30 to 40 individuals per species), this was done for three individuals, categorized in three different diameter classes (small, medium and large). In addition, we also determined WD for species that were not in the destructive analysis but that did occur in our six permanent plots. For these species, two wood core samples were collected at opposite positions at DBH of a standing tree with a DBH close to the mean DBH for that species in the plots.

Each wood core sample was stored in a plastic tube and covered in a plastic bag to avoid water loss, and taken to the Wood Science Laboratory. Fresh volume of each sample was calculated by the formula (π/4)*d^2^*L (where L was the total length and d the mean diameter of the wood core) [23]. Samples were dried at 90°C to constant mass. WD was determined by dividing dry mass by its fresh volume. The WD value of the species was calculated as the mean of the density of the wood core samples.

### 2.5 Data analysis

#### 2.5.1 Tree species functional group

The Vietnamese Forestry Service distinguishes five wood density classes: class I (WD ≤ 0.50 g cm^-3^), class II (0.51 - ≤0.65 g cm^-3^), class III (0.66 - ≤0.80 g cm^-3^) class IV (0.81 - ≤0.95 g cm^-3^) and class V (>0.95 g cm^-3^). These wood density classes are associated with wood quality and the Vietnamese logging regulation [24,25]. Class V was not found in our plots, thus we considered classes I-IV as representing four functional groups.

#### 2.5.2 Allometric equations

For aboveground biomass (AGB) several common equations were tested to develop allometric models that relate the geometric measures (DBH, H and WD) to aboveground biomass (AGB). First, we established equations in which individuals of all species were lumped together (multi-functional group model, denoted as FG-aggregated model hereafter). Second, we developed equations for each individual functional group separately (FG-specific models, hereafter). We tested eight general equations (found in literature) relating AGB to DBH, H and/or WD (see below).

In the case of root biomass (RB), we used the same general equations but added a ninth equation that relates RB to AGB. This builds on the common practice of estimating RB from AGB estimates (e.g. IPCC 2006 [17]). For RB we only developed an FG-aggregated model as there were not enough sample trees to develop FG-specific models.

Several equations are commonly used to develop allometric models for AGB and RB.

By Brown et al. [26] and Brown [1] (pan-tropical):

ln(B)=a+bln(DBH) (1)

$$\text{ln}(B)\;=\;a+b\;\text{ln}(DBH)+b_{1}\;\text{ln}{\left(DBH\right)}^{2}$$ (2)

By Nelson et al. [27] (central Amazon):

ln(B)=a+bln(DBH)+dln(H) (3)

By Chave et al. [3] (pan-tropical):

ln(B)=a+bln(DBH)+cln(WD)+dln(H) (4)

$$\text{ln}(B)\;=\;a+b\;\text{ln}(DBH)+e{\left(\text{ln}(DBH)\right)}^{2}+f{\left(\text{ln}(DBH)\right)}^{3}+c\;\text{ln}(WD)$$ (5)

$$\text{ln}(B)\;=\;a+g\;\text{ln}(DBH^{2}HWD)$$ (6)

By Djomo et al. [28] (tropical Africa):

ln(B)=a+bln(DBH)+cln(WD) (7)

$$\text{ln}(B)\;=\;a+h\;\text{ln}(DBH^{2}\;H)+c\;\text{ln}(WD)$$ (8)

with *B* being *AGB* or *RB*.

An additional equation for estimating root biomass from Lima et al. [2] (Amazonian forests) was used:

ln(RB)=a+bln(AGB) (9)

Following the method of Djomo et al. [28], first we developed equations with only DBH as independent variable. Later, either H or WD was added. Finally, both H and WD were added as independent variables. The ordinary least squares method was used to fit the equations.

All above-mentioned equations relate the natural logarithm of AGB to the natural logarithms of DBH, H and WD. This however introduces a systematic bias in the original biomass estimation. Therefore, back-transformed AGB estimates were adjusted by the correction factor (CF), as defined by $CF\;=\;exp\;\left(\frac{RSE^{2}}{2}\right)$, with RSE indicating the residual standard errors of the estimate [3,29].

#### 2.5.3 Model selection

To select the best fit model, the following statistical indicators were considered:

1. The proportion of variance explained by the model (adjusted R^2^ for the number of predictor variables).
2. The residual standard errors of estimation (RSE) calculated as the square root of the residual sum of squares divided by the degrees of freedom (df) of an estimate. The df was calculated as the number of observations minus the number of predictor variables. The lower RSE, the better the regression model fits [3].
3. Average standard error (S%) calculated by the formula: $\text{S}\%=\frac{100}{n}\sum_{i=1}^{n}\frac{\left|\hat{y}i-yi\right|}{yi}$ where: n is the number of observations; *yi* is the observed dried mass of tree i; $\hat{y}i$is the predicted dried mass of tree i. S% indicates the difference between the observed and the predicted value. The best fit regression is the one with the lowest S% [3,6].
4. Akaike Information Criterion (AIC) is calculated by the formula *AIC* = 2*p* − 2 ln(*L*), where p is the number of parameters in the model and L is the likelihood of the fitted model [3,6,28]. The best model is the one with the lowest AIC.

#### 2.5.4 Comparing FG-aggregated with FG-specific models and local with regional and pan-tropical models

We conducted paired t-tests to compare the best fit FG-aggregated and FG-specific models in estimating biomass of the destructive sample trees and to determine whether predictions by the two types of models were significantly different.

We also compared S% between observed and predicted AGB of the destructive sample trees made by our local model (FG-aggregated), and previously developed regional and pan-tropical models in a similar fashion. Finally, we compared S% between estimates of our local FG-aggregated models and regional and pan-tropical models at the plot level.

All statistical analyses were performed by the IBM SPSS 21.0.

## Results

### 3.1 The functional group of the sample trees

The mean value of wood density of all sample species was 0.63 ±0.008 g cm^-3^ with a range of 0.33–0.89 g cm^-3^. The range of DBH was similar in each class with the exception of WD class III, for which we appeared to have sampled somewhat smaller trees than in the other classes (Table 1).

**Table 1:** Number and size measures of trees that were used in the destructive measurements categorized per wood density (WD) class.

Functional group	Number of individuals (species)	WD (g cm^-3^)	DBH (cm)	H (m)	AGB (kg)
WD I	61(12)	0.45±0.01	47.9±3.5	25.6±0.9	2155±278
WD II	119(20)	0.58±0.01	46.6±2.5	24.8±0.7	255±230
WD III	55(10)	0.70±0.01	37.8±3.2	22.8±1.0	1789±281
WD IV	65(3)	0.85±0.01	51.6±4.3	25.2±1.2	2938±389

WD I: ≤0.50 g cm^-3^, WD II: 0.51-≤0.65 g cm^-3^, WD III: 0.66-≤0.80 g cm^-3^, WD IV: >0.81 g cm^-3^. DBH is diameter at breast height, H is tree height, AGB is above ground biomass (mean values and standard errors of the mean).

### 3.2 Allometric equations

#### 3.2.1 Allometric equations for the FG-aggregated model

Eight common models for moist forest were fitted to our data (Table 2). The adjusted R^2^ of all regressions ranged from 0.981 to 0.986. The lowest adjusted R^2^ was recorded for model 1, while model 8, which included DBH, H and WD as independent variables, exhibited the highest adjusted R^2^ and the lowest values for RSE, AIC and S%. The adjusted R^2^ and coefficient b, which indicates the linear effect of ln(*DBH*) on ln(*AGB*), were significant (p<0.001) in all eight models, indicating DBH to be a consistent predictor of AGB. Coefficient c, which represents the effect of ln(*WD*) on ln(*AGB*), was significant in models 4, 5, 7 and 8 (p<0.001) indicating that WD was also a good predictor of AGB. Coefficient d indicates that H was also a good predictor of AGB when used together with DBH (in models 3 and 4) (analysis for significance of coefficients not shown). As model 8 gave the best fit, it was selected for predicting AGB in our study as follows:

$$\text{ln}(AGB)\;=-3.081+0.966\;\text{ln}(DBH^{2}H)+0.305\;\text{ln}(WD)$$ (10)

The equation (after log-transformation) was corrected by a correction factor (CF) that eliminated the systematic bias produced by the logarithms transformation [30]. Therefore the final equation was:

$$AGB\;=\;exp(-3,051+0.966\;\text{ln}(DBH^{2}H)+3.05\;\text{ln}(WD))$$ (11)

**Table 2:** Model description of above ground biomass (AGB) estimates for the FG aggregated model.

No	Model	Adjusted R^2^	RSE	AIC	S%	CF
1	ln(*AGB*) = *a* + *b*ln(*DBH*)	0.981	0.287	-747.4	23.5	1.042
2	ln(*AGB*) = *a* + *b*ln(*DBH*) + *b1*(ln(*DBH)*)^2^	0.983	0.270	-785.3	21.6	1.037
3	ln(*AGB*) = *a* + *b*ln(*DBH*) + *d*ln(*H*)	0.985	0.254	-820.9	20.6	1.032
4	ln(*AGB*) = *a* + *b*ln(*DBH*) + *c*ln(*WD*) + *d*ln(*H*)	0.986	0.245	-840.8	19.8	1.030
5	ln(*AGB*) = *a* + *b*ln(*DBH*) + *e*(ln(*DBH*)^2^ + *f*(ln(*DBH*))^3^ + *c*ln(*WD*)	0.985	0.254	-823.8	19.9	1.032
6	ln(*AGB*) = *a* + *g*ln(*DBH*^*2*^*HWD*)	0.981	0.287	-744.3	23.9	1.040
7	ln(*AGB*) = *a* + *b*ln(*DBH*) + *c*ln(*WD*)	0.982	0.283	-755.1	23.3	1.040
8	ln(*AGB*) = *a* + *h*ln(*DBH*^*2*^*H*) + *c*ln(*WD*)	0.986	0.245	-841.2	19.8	1.030

The results are significant at a 95% confidence interval. DBH, H, WD indicate stem diameter at breast height, tree height and wood density, respectively. RSE: residual standard error of the estimate, AIC: Akaike Information Criterion, S%: average standard error of the estimate, CF: correction factor.

#### 3.2.2 Allometric equations for different functional groups (FG)

Next we compared the different allometric equations for the functional type specific models (Table 3).

**Table 3:** Model description of above ground biomass (AGB) estimates of four functional groups.

Model	Group I				Group II				Group III				Group IV			
	Adjusted R^2^	RSE	AIC	S%	Adjusted R^2^	RSE	AIC	S%	Adjusted R^2^	RSE	AIC	S%	Adjusted R^2^	RSE	AIC	S%
1	0.980	0.279	-156.4	22.4	0.982	0.268	-313.8	21.6	0.983	0.253	-152.2	21.1	0.990	0.235	-188.8	17.8
2	0.982	0.270	-161.4	21.3	0.986	0.239	-341.7	18.7	0.986	0.226	-165.2	17.5	0.992	0.216	-200.7	16.1
3	0.984	0.254	-170.9	20.0	0.988	0.221	-357.6	17.5	0.991	0.186	-183.9	14.4	0.993	0.199	-208.6	15.5
4 (-)	0.984	0.254	-162.0	19.7	0.988	0.220	-354.6	17.4	0.991	0.187	-179.0	14.2	0.993	0.200	-204.4	15.5
5 (-)	0.983	0.264	-163.5	19.8	0.986	0.240	-340.6	20.0	0.986	0.230	-162.6	17.4	0.993	0.206	-206.3	14.8
6	0.984	0.264	-159.4	19.6	0.987	0.226	-346.2	17.8	0.991	0.186	-177.5	14.1	0.993	0.198	-203.4	15.7
7 (-)	0.980	0.280	-154.2	22.8	0.982	0.269	-311.3	21.5	0.983	0.253	-150.2	20.7	0.990	0.235	-186.8	17.4
8 (-)	0.984	0.255	-160.8	19.8	0.988	0.220	-354.5	17.3	0.991	0.186	-178.8	14.1	0.993	0.199	-203.6	15.4

The results are significant at a 95% confidence interval. Functional groups are wood density (WD) classes I–IV (group I: WD ≤0.50 g cm^-3^, group II: WD 0.51-≤0.65 g cm^-3^, group III: WD 0.66-≤0.80 g cm^-3^, group IV: WD>0.81 g cm^-3^). RSE: residual standard errors of the estimate, AIC: Akaike Information Criterion, S%: average standard error of the estimate, (-) indicates the model has a predictor that was not significantly different from 0.

*Functional group I (wood density ≤0*.*5 g cm*^*-3*^*)*: The highest adjusted R^2^ was recorded for models 3, 4, 6 and 8 (0.984). Coefficient d was significant only in models 3 and 4 (p<0.001). Together with DBH, predictor H had an important role in the estimate. Model 3 was selected based on the highest adjusted R^2^ (0.984) and the lowest RSE (0.254) and AIC (-170.9). Therefore the best model for functional group I was:

ln(AGB)=−3.587+2.141ln(DBH)+0.773ln(H) (12)

*Functional group II (wood density 0*.*51- ≤0*.*65 g cm*^*-3*^*)*: The highest adjusted R^2^ was recorded for models 3, 4 and 8 (0.988). Coefficient c was not significant in models 4 and 8, therefore these models were rejected. The lowest AIC was found in model 3 (-357.6). We therefore chose model 3 yielding:

ln(AGB)=−3.406+1.958ln(DBH)+1.017ln(H) (13)

*Group III (wood density 0*.*66- ≤0*.*8 g cm*^*-3*^*)*: The highest adjusted R^2^ value was found for models 3, 4, 6 and 8 (0.991). The RSE and AIC values were lowest for model 3; therefore, model 3 was selected for functional group III:

ln(AGB)=−3.161+2.005ln(DBH)+0.896ln(H) (14)

*Group IV (wood density >0*.*81 g cm*^*-3*^*)*: The highest adjusted R^2^ value was found for models 3, 4, 5, 6 and 8 (0.993). The lowest value of RSE was given by model 6, however the lowest AIC was given by model 3. The S% of model 3 was 15.5% compared to 15.7% of model 6, hence model 3 was selected.

ln(AGB)=−2.567+1.945ln(DBH)+0.734ln(H) (15)

#### 3.2.3 Aboveground biomass predicted by the FG-aggregated model and FG-specific models

We compared the predictions made with the aggregated model to the combined prediction of the FG-specific models (Table 4). The average standard error (S%) in the estimates of the aggregated FG-model was 19.8% compared to 17.0% for the FG-specific models. The differences in AGB estimates between the two models were analyzed by comparing the prediction for the mean AGB of all trees in the sample to the mean observed values, using paired samples t-tests at a 95% confidence interval. Predictions of neither model were significantly different from the observed values (p>0.05 for pairs 1 and 2). Similarly, the two model predictions of both tree level and plot level AGB (pair 3 and pair 4) values were not significantly different from each other.

**Table 4:** Paired t-tests for the mean differences between observed and predicted above ground biomass (AGB) of destructively sampled trees.

Pairs	Mean differences	Std. Deviation	Std. Error Mean	S%	t	P
(1): Observed vs. Predicted AGB by FG aggregated model	-92.41	920.6	55.30	19.8	-1.67	0.096
(2): Observed vs Predicted AGB by FG specific models	-64.36	932.1	39.02	17.0	-1.64	0.100
(3): Predicted AGB by FG aggregated vs FG specific models	28.05	753.8	34.12	10.7	0.82	0.412
(4): Predicted AGB (plots level) by FG aggregated vs FG specific models	-6.01	9.6	3.61	2.1	-1.66	0.157

Tests were made with the FG-aggregated (pair 1) and FG-specific models (pair 2), and for the mean differences between these two models in estimates of mean tree biomass (pair 3) and mean plot level AGB for the six plots (pair 4) in this study. The results are significant at a 95% confidence interval. Mean differences, Standard (Std) deviation and Standard error of the mean in pair 1, 2 and 3 are in kg; in pair 4 this is in ton per ha. S% indicates the average standard error of the estimate.

#### 3.2.4 AGB estimates at tree and plot level by the FG-aggregated model and regional and pan-tropical models

We determined the extent to which the estimates of the AGB of sample trees and the total AGB in our permanent plots calculated with the local FG-aggregated model developed here, differed from those made by a number of regional and pan-tropical models (Table 5). These models included both global models for tropical moist forest [1,3] and regional models developed in other parts of SE Asia [8,16].

**Table 5:** Model description for aboveground biomass (AGB) estimates.

No	Models	Forest type	N	DBH (cm)	WD (g cm^-3^)	Valid Region
1	AGB = exp(-2.289+2.649ln(*DBH*) -0.021ln(*DBH*^2^))	M	170	5.0–148.0		Global
2	AGB = 0.0509*WDDBH*^2^*H*	M	2410	5.0–156.0		Global
3	AGB = 0.11*WDDBH*^2.62^	S	29	7.6–48.1	0.60	Indonesia
4	AGB = 0.0829*DBH*^2.43^	S	136	0.1–28.7	0.35	Malaysia
5	AGB = exp(-3.051+0.966ln(*DBH*^*2*^*H*) +0.305ln(*WD*))	M	300	1.8–115.0	0.63	Vietnam

Models: (1) Brown [1], (2) Chave et al. [3], (3) Ketterings et al. [16], (4) Kenzo et al. [8] and (5) FG-aggregated model (this study). DBH, H, and WD indicate stem diameter at breast height, tree height and wood density, respectively. Forest type: M is tropical moist mature forest, S is tropical secondary forest. N is the number of sample trees. DBH is the diameter range of sample trees and WD is the mean wood density of sample trees used to develop the model.

**Fig 1:**
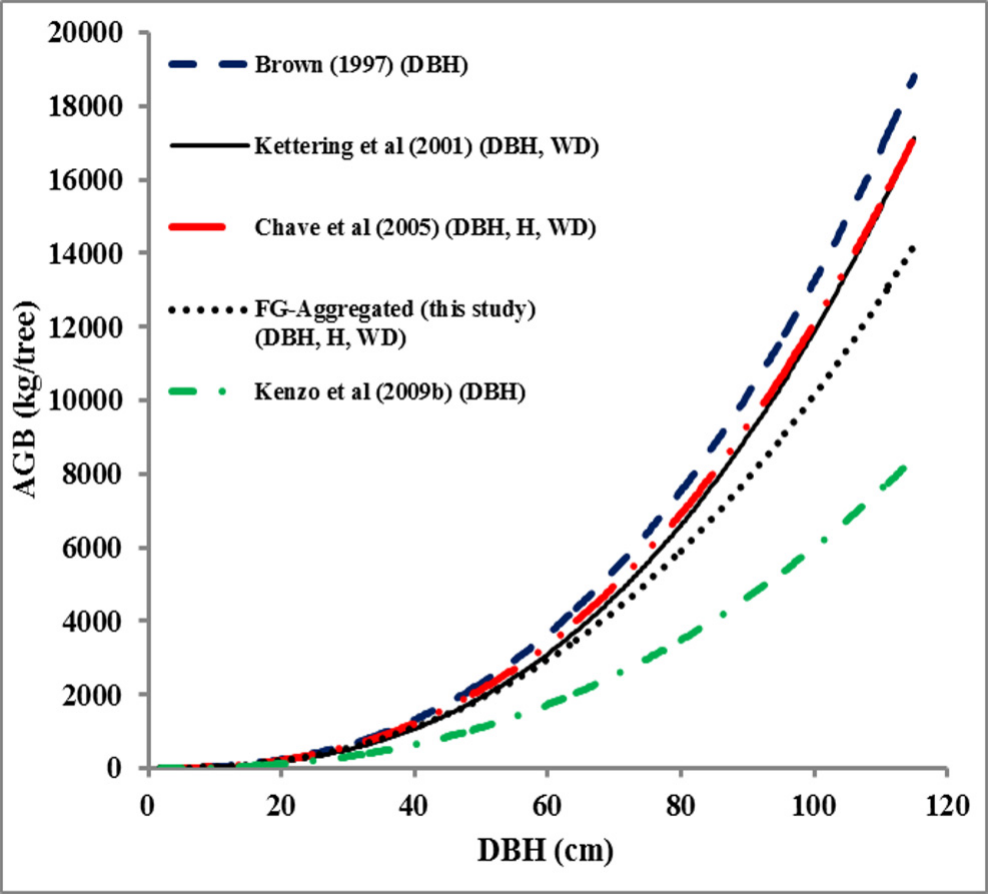
The predicted above ground biomass (AGB) of the sample trees (kg tree^-1^) made by our FG-aggregated model and by a number of regional and pan-tropical models. Model used were Brown [1], Ketterings et al. [16], Chave et al. [3], and Kenzo et al. [8], as a function of DBH. The parameters in the brackets indicate the predictors that were used in the model.

**Fig 2:**
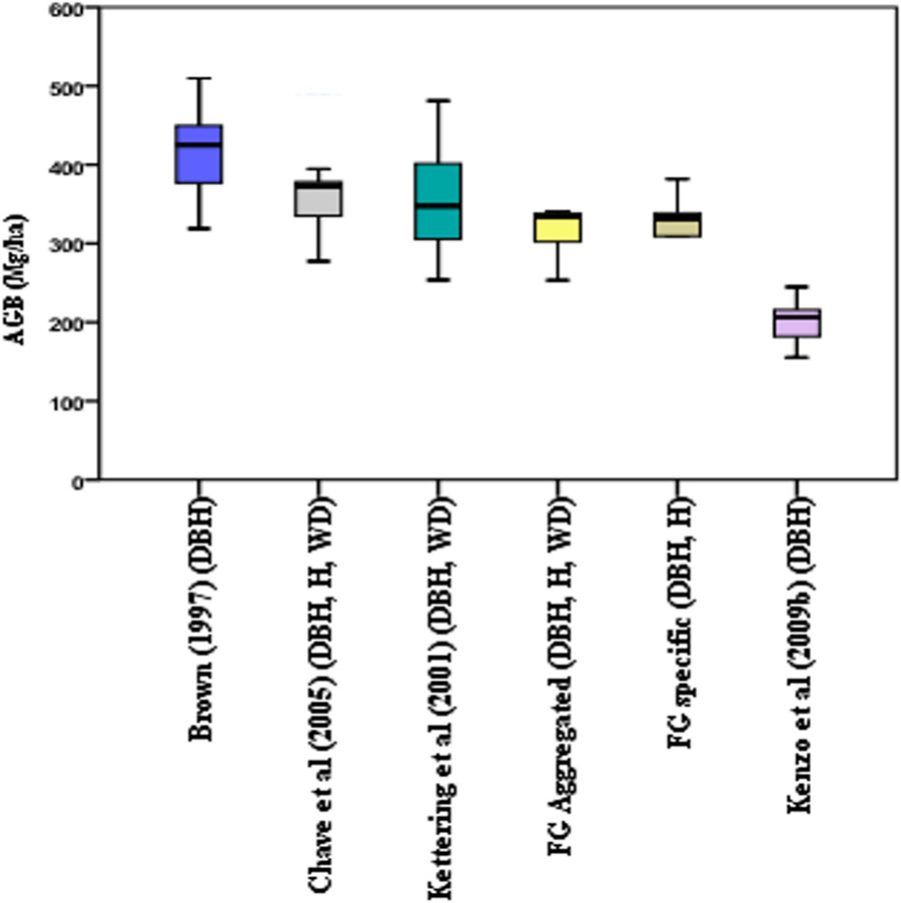
Boxplots showing the mean predicted AGB (Mg ha^-1^) of six plots. AGB was predicted by Brown [1], Chave et al. [3], Ketterings et al. [16], Chave et al. [3], our models (both FG-aggregated and FG-specific models) and Kenzo et al. [8]. The parameters in the brackets indicate the predictors that were used in the model.

The AGB values of the sample trees (Fig 1) were considerably overestimated by the models of Brown [1], Ketterings et al. [16] and Chave et al. [3] (i.e., S% value of 34.3%, 31.2% and 29.2%, respectively), but were considerably underestimated (S% value of 38.4%) by the model of Kenzo et al. [8]. The t-tests showed that there were significant differences of predicted AGB between our FG-aggregated model and the regional and pan-tropical models (p<0.05). Predictions of the plot-level AGB (Fig 2) made with the various models differed considerably; the AGB estimated by the model of Brown [1] was more than twice the AGB predicted by the model of Kenzo et al. [8]. When compared to our aggregated model, the model by Brown [1] resulted in a 27.4% higher estimate of plot level AGB followed by the model by Chave et al. [3] (13.9% higher estimate) and the model by Ketterings et al. [16] (8.0% higher estimate). Conversely, the model by Kenzo et al. [8] resulted in a 38.3% underestimation. All these differences were significant (t-test P<0.05) except for the model by Ketterings et al. [16].

#### 3.3 The development of equations for root biomass

We tested several allometric equations that relate root biomass (RB) to the aboveground size measures such as DBH, H and WD. In addition we also conducted regressions between RB and AGB (Table 6).

**Table 6:** Model description of root biomass (RB) estimates.

No	Model	Adjusted R^2^	RSE	AIC	S%	CF
1	ln(*RB*) = *a* + *b*ln(*DBH*)	0.790	0.391	-75.16	30.7	1.079
2 (-)	ln(*RB*) = *a* + *b*ln(*DBH*) + *e*(ln(*DBH*))^2^	0.785	0.396	-75.16.	30.8	1.081
3 (-)	ln(*RB*) = *a* + *b*ln(*DBH*) + *d*ln(*H*)	0.789	0.392	-73.93	30.7	1.079
4 (-)	ln(*RB*) = *a* + *b*ln(*DBH*) + *c*ln(*WD*) + *d*ln(*H*)	0.856	0.324	-88.26	22.2	1.053
5 (-)	ln(*RB*) = *a* + *b*ln(*DBH*) + *e*(ln(*DBH*))^2^ + *f*(ln(*DBH*))^3^ + *c*ln(*WD*)	0.854	0.326	-87.73	22.5	1.054
6	ln(*RB*) = *a* + *b*ln(*DBH*^*2*^*HWD*)	0.854	0.326	-85.48	23.3	1.054
7	ln(*RB*) = *a* + *b*ln(*DBH*) + *c*ln(*WD*)	0.857	0.323	-89.52	22.8	1.053
8	ln(*RB*) = *a* + *b*ln(*DBH*^*2*^*H*) + *c*ln(*WD*)	0.854	0.326	-86.61	22.3	1.054
9	ln(*RB*) = *a* + *b*ln(*AGB*)	0.860	0.322	-90.6	25.5	1.053

DBH, H, WD and AGB are predictors of the models and indicate stem diameter at breast height, tree height, wood density and above ground biomass, respectively. The results are significant at a 95% confidence interval. RSE: residual standard errors of estimate, AIC: Akaike Information Criterion, S%: average standard error of the estimate, CF: correction factor, (-) indicates the model has a predictor that was not significantly different from 0.

The highest adjusted R^2^ and lowest AIC and RSE were recorded when RB was plotted against AGB (model 9). Model 7 relates RB to DBH and WD and was second best in this respect (Table 6). The lowest average standard error (S%) was recorded for model 4 that relates RB to DBH, H and WD, however, coefficient d, indicating the effect of H, was not significant; therefore, this model is essentially similar to model 7 with DBH and WD as predictors. Models 3 and 8 had a lower adjusted R^2^ and higher RSE and AIC values compared to models 7 and 9. Moreover, tree height (H) is often difficult to measure accurately in forests [3]. Models 1 and 2 showed relatively weak predictive power with only DBH as input. Model 9 is the best selection if only AGB data are available. The equation after back log-transformation is:

RB=exp(−0.804+0.823ln(AGB)) (16)

When DBH and WD are available, model 7 would be recommended:

ln(RB)=−1.703+1.934ln(DBH)+1.06ln(WD) (17)

The equation after back log-transformation is as follows:

RB=exp(−1.651+1.934ln(DBH)+1.06ln(WD)) (18)

Since WD data are available for our study, we selected model 7 for further analyses.

**Fig 3:**
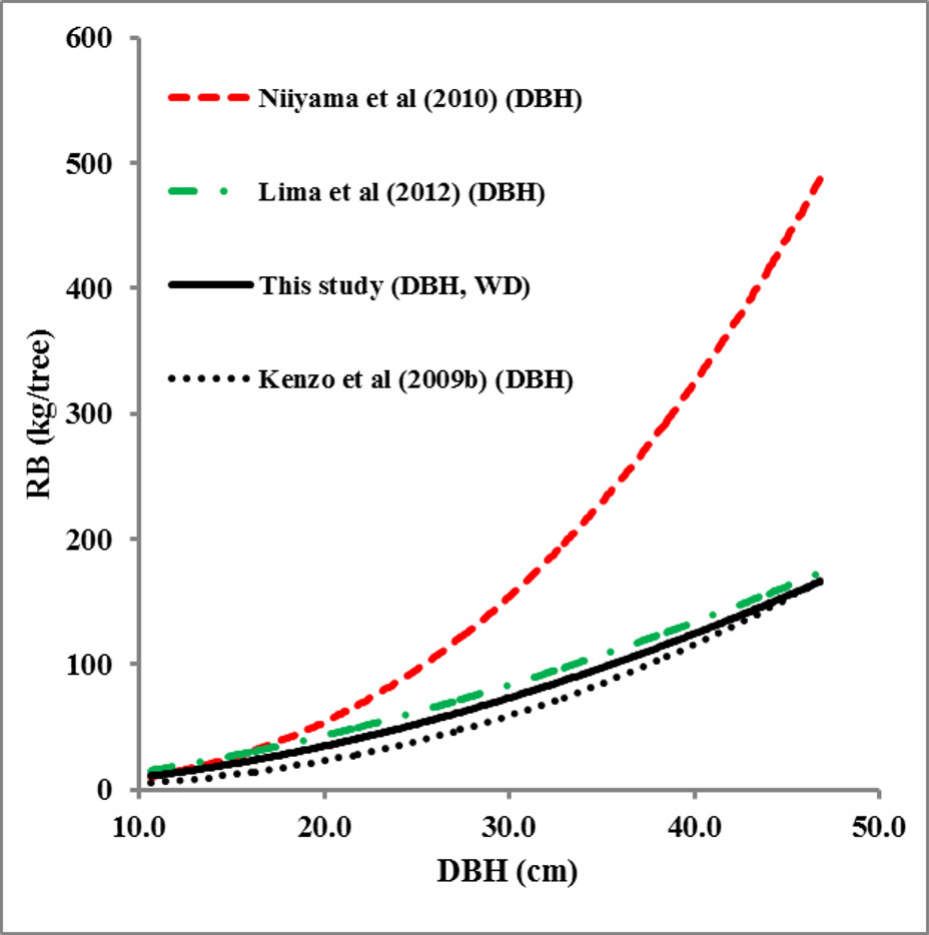
Root biomass (RB) of sample trees. Root biomass (kg tree ^-1^) was predicted by the model of this study and the models of Niiyama et al. [20], Lima et al. [2] and Kenzo et al. [8]. The parameters in the brackets indicate the predictors that were used in the model.

**Fig 4:**
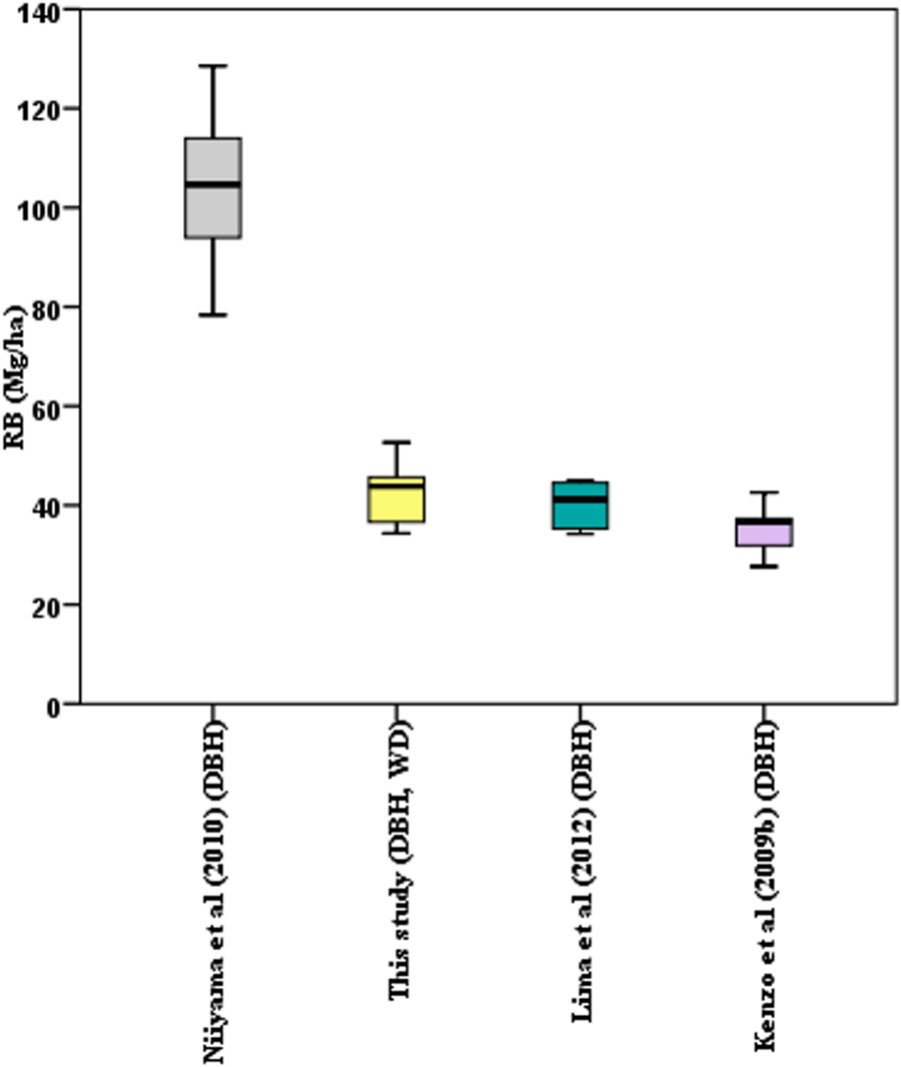
Boxplots showing the value of root biomass in six plots. Root biomass (ton ha^-1^) was predicted by Niiyama et al. [20], this study, Lima et al. [2] and Kenzo et al. [8]. The parameters in the brackets indicate the number of predictors in the model.

Next, we compared predictions of the mean root biomass in our six permanent plots by model 7 against a set of regional and pan-tropical models (Fig 3), i.e Kenzo et al. [8], Niiyama et al. [20] and Lima et al. [2]. Two of the foreign model estimates differed considerably from ours, with the model of Niiyama et al. [20] resulting in a nearly 140% higher estimate and the model of Kenzo et al. [8] in a 18% lower estimate (Fig 4). The model of Lima et al. [2] resulted in a 7% lower estimate and was not significantly different from ours (P >0.05).

The average ratio between root biomass (RB) and aboveground biomass (AGB) of all sampled trees was 0.19 (range 0.09–0.43) and did not differ among the six plots in our study site (p<0.05).

## Discussion

The choice of allometric models represents a major source of uncertainty in the estimation of biomass in tropical forests. Particularly the balance between model accuracy on the one hand, and the effort involved in model parameterization on the other, needs to be carefully considered. Here we considered three aspects related to model choice: (i) the number and descriptive parameters to be used, (ii) the degree to which models should be specific at the level of tree functional types and (iii) use of local versus regional and pan-tropical models. We did this for both above- and belowground biomass. We showed that the functional group aggregated local model with three predictors (DBH, H and WD) is the best choice for predicting AGB in the forest of this study. There were considerable differences in biomass estimates between our models and extant pan-tropical models, confirming existing concern about the applicability of pan-tropical models at the plot level. Parameterization of local models, especially those for RB, remains an important issue of concern. We will discuss our findings in more detail below.

### 4.1 FG-aggregated species model

We compared eight allometric models to test the predictive value of three descriptive parameters, i.e. diameter (DBH), wood density (WD) and height (H) for AGB. Of these models the one using DBH (model 1) as the only descriptive parameter provided the lowest accuracy. Adding either H or WD improved model predictions but the effect of WD was larger, while adding both WD *and* H resulted in a slight further improvement. These results support the view that in addition to DBH, H and WD are important predictors of aboveground biomass in tropical forest [3,28,31]. For further comparative analyses we thus chose a model that combined all three parameters (model 3).

Several studies based on both local and global data sets have emphasized the importance of WD as a predictor of AGB for tropical forest [3,5,9,28,32], especially for non-pioneer trees [33]. WD of tropical forests may differ considerably between regions and strongly depends on the successional stage of forests [30]. This variation in WD across forests makes its use as a predictor essential; if not, a model developed for a lower WD than where it is applied leads to underestimation of biomass and vice versa. The average WD of the sample trees that we used to develop our allometric equations was 0.63 g cm^-3^, which is close to the value for a tropical moist forest in Cameroon and Ghana [28,33] and somewhat higher than the value in a secondary tropical forest in Indonesia [16]. Further complications arise if WD is not uniformly distributed over size, e.g. if larger species have higher or lower WD than smaller ones [30]. In our study there was a positive correlation between the mean tree size and WD of a species (results not shown), thus averaging WD for all species or across groups of species would tend to lead to underestimations of AGB.

In some studies WD did not appear to significantly improve model predictions of AGB, particularly in studies in secondary and *Dipterocarp* forests [7,8] This could be associated with the fact that the dominant trees (i.e., those that account for the largest fraction of AGB) in such forests tend to exhibit a narrow range in wood density (e.g the range of WD was 0.29–0.53 g cm^-3^ in the study of Kenzo et al. [8] in a young tropical secondary forest with many pioneer species).

In our model, H is a significant predictor, but it is difficult to measure accurately because canopy layers hide tree tops in the forest. This is a common phenomenon especially in tropical forest. In order to increase the quality of the measurement of H, accurate instruments, such as laser altimeters, should be used to determine tree heights in tropical forest.

### 4.2 Functional group-specific model

As noted, multi-species models may entail inaccuracies due to the vast different allometric traits that may exist among species, but development of species-specific models may not be feasible in tropical forests where the number of species are too large and individuals per species too few to provide solid parameterization [4]. The use of a functional type specific model, whereby species are grouped, could be an alternative. We assessed the extent to which a functional type specific allometric model (FG specific), i.e., one that is parameterized per functional type defined by wood density, resulted in more accurate estimates than the aggregated model.

The equations were tested on the trees from four WD density classes (i.e., functional types) resulting in FG-specific models that provided better fits to sample trees than the aggregated model, i.e., as reflected in their higher adjusted R^2^ and lower standard error in the estimate of the AGB of the sample trees (Table 3).

We also found that WD was not a significant predictor of AGB in any of the FG-specific models. In each functional type, Pearson correlations showed that AGB was more strongly related with DBH and H than with WD. This smaller role of WD can be attributed to the fact that the range in WD within functional types was smaller by definition. These findings are consistent with another study showing that the range in WD density as well as its role in allometric equations was much less prominent in genus specific models than in multi-species ones [6].

**Fig 5:**
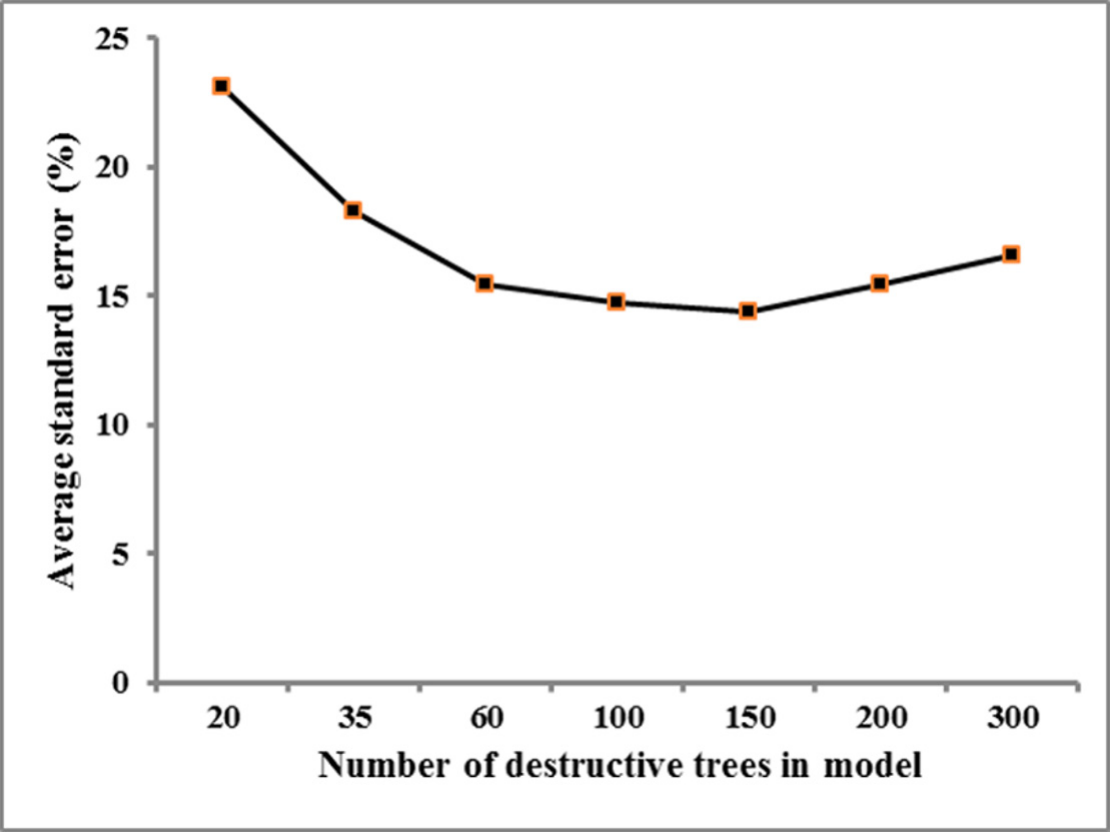
The average standard error (S%) in the estimate of total AGB of 30 selected trees. Each square was the average standard error (S%) of the estimate (Y axis) given by the model that was developed by a certain number of destructive trees i.e., 20, 35, 60, … and 300 trees, respectively (X axis).

Even though the FG-specific model provided a better fit and an AGB estimate with a smaller error than the FG-aggregated model, there were no significant differences of accuracy in AGB estimates for both sample trees and plots between the two models. This result is consistent with e.g. Fayolle et al. [5] in that species-specific models do not always provide better fits than multi-species models. This leaves the question as to why, in spite of its greater detail, the FG-specific model was not significantly more accurate? Several factors may have contributed to this. Firstly, in the FG-specific models, individual regressions were done per WD class and thus the number of trees in each of the samples was much smaller (55–119, Table 1) than in the FG-aggregated model (300). Limitations on sample size have been forwarded as an important constraint on the use of more specific models [9,30]. However, we conducted an analysis to determine the sensitivity of the standard error in the AGB estimate by the FG-aggregated model, for sample size (Fig 5), and found the error not to be very sensitive over the range 50–300 individuals.

Secondly, in our study functional types were defined according to WD which was also added as a predictor in the aggregated model, and thus much of the variation between functional types was already accounted for. But tree functional types may differ in other respects as well. For example both theoretical models and data suggest trees of high WD to be more slender (greater height per DBH) than low WD trees [34,35]. Nevertheless, tree allometry is probably more strongly determined by environmental factors than by species or functional type [4], suggesting that such variation in slenderness across functional types it is probably small. To summarize, our results indicate that at least in our forest, the use of an aggregated model is merited. However, a functional type specific model could be more efficient than an aggregated one in situations where one does not know the exact WD of individual species but species are categorized in WD classes.

### 4.3 Local models versus regional and pan-tropical models

Estimates of the AGB of sample trees made by regional and pan-tropical models differed considerably from the observed values in our study. Similarly, large differences were observed when plot level AGB estimates by regional and pan-tropical models were compared to the plot level estimates made with our local aggregated model. Particularly large deviations were found for the models that were based on DBH alone, i.e., Brown [1] resulted in more than 25% overestimations while Kenzo et al. [8] in almost 40% underestimation. The deviations were smaller for regional and pan-tropical models that included WD; e.g. the much-used Chave et al. [3] model resulted in a 14% overestimation of plot level AGB, and a 30% higher estimate of sample trees AGB.

The use of regional and pan-tropical models implies that one does not know their accuracy beforehand, that is, if they are not locally checked. This raises questions regarding the consistency of the margins of error that we observed. That is, error consistency or at least predictability (e.g. linked to forest type or region), could render regional and pan-tropical models more useful. Unfortunately, thus far, local-regional and pan-tropical model comparisons are still relatively rare, and mostly restricted to the pan-tropical models of Brown [1] and Chave et al. [3]. Considerable (at least up to two-fold) overestimations of AGB (50–65%) of Brown [1] model have been observed in a number of studies [6–8]. Such overestimation may arise when models are applied to trees that tend to be smaller than those for which model was parameterized [9]. However, in our study, the diameter of the sample trees that were used to develop allometric models are close to those diameters in Brown [1] and Chave et al. [3] (Table 5), while an overestimation was still found. For the model by Chave et al. [3] the results are quite variable ranging from up to two-fold overestimations [2,6] to 30–40% underestimations (e.g. Henry et al. [33]) of AGB. Other studies reported reasonably close fits (e.g. Fayolle et al. [5] and citations therein) or size dependent fits whereby Chave et al. [3] underestimated AGB of low density plots and overestimated AGB of high density plots [9]. It could be argued that Kenzo et al. [8] underestimated our biomass data because their model was developed for an early successional forest with relative low wood density. However, the Kenzo et al. [8] model also underestimated the AGB of our group I (lowest WD) alone (data not shown), indicating that other factors contributed to the mismatch.

These comparisons indicate two points. First, there is a reasonably consistent pattern that inclusion of WD in regional and pan-tropical models can lead to more accurate estimates. This is important in view of the of the fact that current IPCC guidelines still recommend the use of the Brown [1] model that is based on only DBH [17,36]. Second, even inclusion of WD in regional and pan-tropical models gives rather inconsistent results in terms of accuracy and the direction of errors. The important advantage of regional and pan-tropical models however is that they are usually based on a much larger data sets, e.g.> 2000 trees as in the case of Chave et al. [3] that span various regions. Local models are often based on less than 100 trees which may be problematic [4,30]. Moreover at larger scales, errors in estimates from regional and pan-tropical models can cancel each other out [9]. The inconsistency in the performance, and the potential for large errors of the Chave et al. [3] model however calls for caution.

We suggest that the selection of allometric models for AGB estimates is very important and depends on the purpose of the estimate or research questions. In small scale plots for instance, to conduct comprehensive estimates of biomass dynamics, model accuracy becomes more important while a representative destructive sample is more easily obtained. In such cases a local model might be more suitable. On the other hand, in large scale studies, e.g. in the case of monitoring REDD+ projects at the landscape or regional level, plot level errors may cancel each other out while the data set required for parameterization might become prohibitively large. Thus, the use of extant regional and pan-tropical models may provide the most rigorous estimates in such cases [9].

We used the same destructive samples (300 trees) for calibration and validation of our local models. This is different from several other studies, e.g Chave et al. [37], Ishihara et al. [38], in which the dataset was split for calibration and validation. In our study, only 45 species were destructively sampled of which 22 species had only one individual; hence, splitting the dataset may thus not be necessary in our case. To support our point we did separate the whole dataset in two parts at random. We used 150 sample trees to calibrate a new FG-aggregated model and the rest (another 150 trees) was used to validate the model, and to compare to regional and pan-tropical models. The S% (the average standard error between observed and estimated AGB of 150 validated destructive samples) was a 31.1%, 31,7% and 29.5% overestimation of the models by Brown [1], Ketterings et al. [16] and Chave et al. [3] respectively, and a 39.8% underestimation of the model of Kenzo et al. [8]. The S% was similar (19.8% vs 19.6%) between the original FG-aggregated model (model developed by 300 destructive trees- model 11) and the local FG-aggregated model developed by using 150 destructive trees. The paired samples t-tests at a 95% confidence interval showed that there were significant differences between observed and predicted AGB of sample trees (150 trees) made by global and regional models (p<0.05), but not significantly different from the estimates made by the FG-aggregated model developed by using 150 destructive trees) (p>0.05).

We suggest that, to develop a local model, splitting the data for calibration and validation may not be necessary when the destructive tree samples cover all tree sizes found in the forest.

### 4.4 Root biomass models

Compared to AGB, models to estimate root biomass (RB) are rare. Currently, the IPCC [17] advises the use of a root-shoot ratio (RS) of 0.24 to estimate RB in moist tropical forests even though RS values can vary between 0.04–0.33 [1]. We developed several models for a data set of 40 trees (RS range: 0.09–0.43) and compared them to extant regional and pan-tropical models.

Regarding the geometric size measures in the RB models used in our study, we found both DBH and WD to be good predictors of root biomass but H was not significant for all of these models. Nevertheless, Pearson correlation showed that the RB-WD relationship is much less significant than RB–DBH in each regression. The highest adjusted R^2^ and the lowest AIC were given by the model in which RB was estimated as a function of AGB. This was consistent with other findings, e.g Cairns et al. [18], and reflects the strong functional relationship between roots and shoots. Different from our study, several previous studies chose the simplest regression of RB = aDBH^b^ (a and b were coefficients of the regression) to predict RB in both secondary and primary forest, such as Kenzo et al. [8], Niiyama et al. [20] and Lima et al. [2], in part to avoid the need of having to estimate WD and/or H. However, as both RB and AGB are often estimated simultaneously in an attempt to assess total biomass, the choice of parameters to be used in allometric models for RB often depends on the predictors that are used in the AGB model; that is, an additional parameter for the RB model in our study may not be a problem if this parameter needs to be measured anyway.

We found that the mean RS ratio was 0.19 across the six plots in our study site, which is close to 0.18 of *Dipterocarp* forest [20] and much higher than the average of 0.12 in lowland moist forest [1]. Nevertheless, it was lower than the value of 0.24 recommended by the IPCC [17]. Two factors may have influenced our RB estimates. First, we did not estimate the mass of roots that was left behind after excavation. Niiyama et al. [20] estimated this to be 23% of root biomass in their site suggesting that our model might underestimate RB. Interestingly, if we correct our RB for the 23% of root mass loss found by Niiyama et al. [20] the RS in our study would be 0.237, similar to the IPCC [17] recommended value of 0.24. Second, for RB measurements, we sampled trees up to a maximum DBH of about 47 cm while our plots included trees of up to 120 cm. However, RS does not seem to be very size-dependent [19].

Model choice to determine RB may involve a number of considerations in addition to those discussed for AGB. First, as with AGB model choice it depends on the objective of the study. If the objective is to determine whole stand biomass, then accuracy in RB estimates is not as significant as AGB, simply because RB constitutes a relatively smaller part of the total. For instance, in monitoring REDD+ projects, RB is estimated to be 24% of the AGB [17], which in our case possibly leads to an overestimation of about 20% (though as noted this was probably less). At the level of total biomass this would translate into an error of 4–5%. Thus, in our case even the IPCC standard would probably have been a reasonable option. On the other hand, if the focus is on root mass itself, more accuracy is needed. A second issue is the type of model used. As noted, RB models can be categorized as being either based on geometric measurements (as are the AGB models) or based on AGB by assuming a given and fixed root:shoot ratio (RS). The latter is most commonly used (see [17] recommendation), and when locally developed, may provide high accuracy as was shown here. However, the use of foreign RB models based on AGB entails a multiplication of errors: one associated with the model itself (i.e., the assumed RS value) and one associated with errors in the model that estimates AGB.

## Conclusions

The multi-species equation (FG-aggregated species) with three predictors of DBH, WD and H is the best choice to estimate plot level AGB at least at our site. Wood density is an important predictor in allometric equations and improves the quality of the estimate for both above and belowground biomass. This paper further confirms concerns that regional and pan-tropical models based on large data sets may lead to erroneous biomass estimates of plot based biomass. The choice of local or pan-tropical forest models for predicting AGB or RB depends on the scale and purpose of the study. For landscape or regional level biomass estimates, pan-tropical models might be the best solution in terms of cost and accuracy. While this study may contribute to the general debate regarding the development and use of allometric equations for estimating above and belowground biomass, it also adds vital data in this regard for Vietnamese forests for which such methods have not yet been developed.

## Supporting Information

S1 Fig Map of the study site. (TIF)

S1 Table Destructively sampled trees. (XLSX)

S2 Table Summary of destructively sampled species information. (DOCX)
