# Supplementary figures and images for: Allometric Equations for Aboveground and Belowground Biomass Estimations in an Evergreen Forest in Vietnam
Source: PLoS One. 2016 Jun 16;11(6):e0156827. doi: 10.1371/journal.pone.0156827 (PMC4910975; doi:10.1371/journal.pone.0156827)

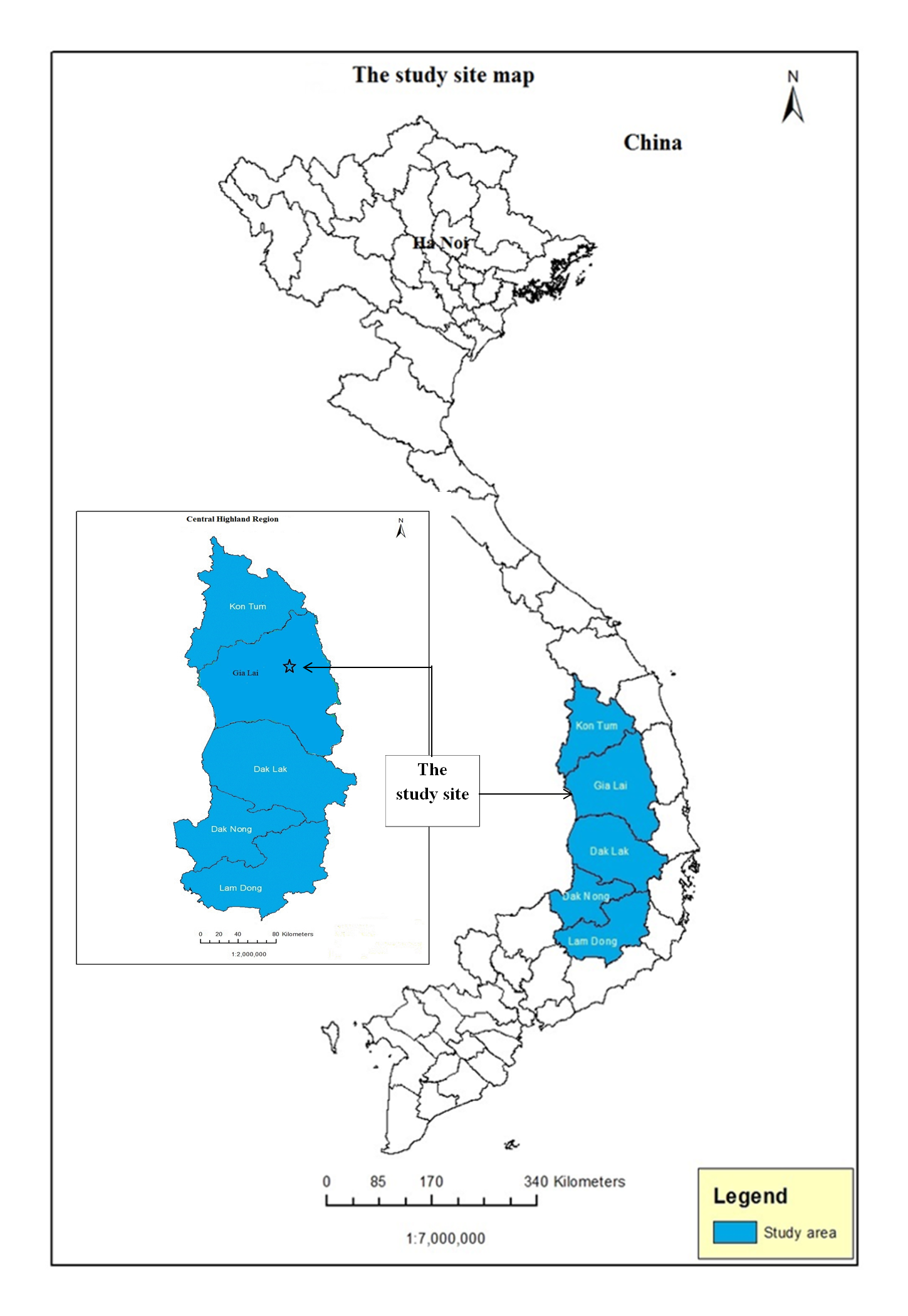

Supplement: S1 Fig — (TIF) [file pone.0156827.s001.tif]
