# Supplementary material for: Allometric Equations for Aboveground and Belowground Biomass Estimations in an Evergreen Forest in Vietnam
Source: PLoS One. 2016 Jun 16;11(6):e0156827. doi: 10.1371/journal.pone.0156827 (PMC4910975; doi:10.1371/journal.pone.0156827)
Supplement: S2 Table — (DOCX) [file pone.0156827.s003.docx]

**Summary of destructively sampled species information**

| No | Species | Family | Number of individuals | DBH range  (cm) | H range  (m) | WD  (gcm^-3^) |
| --- | --- | --- | --- | --- | --- | --- |
| 1 | *Actinodaphne pilosa* | Lauraceae | 1(1) | 21.1 | 23.0 | 0.33 |
| 2 | *Aglaia spectabilis* | Meliaceae | 4 | 29.6-71.0 | 27.0-33.1 | 0.65 |
| 3 | *Aglaia macrocarpa* | Meliaceae | 4(1) | 15.0-76.0 | 13.4-28.5 | 0.59 |
| 4 | *Ailanthus triphuysa* | Simaroubaceae | 1 | 49.0 | 35.5 | 0.45 |
| 5 | *Alangium ridleyi* | Alangiaceae | 1(1) | 30.5 | 26.8 | 0.44 |
| 6 | *Alstonia scholaris* | Apocynaceae | 4 | 62.0-85.0 | 28.0-35.5 | 0.43 |
| 7 | *Antidesma ghasembilla* | Euphorbiaceae | 5 (5) | 11.5-32.5 | 11.8-19.8 | 0.67 |
| 8 | *Baccaurea ramiflora* | Euphorbiaceae | 1(1) | 20.6 | 18.0 | 0.38 |
| 9 | *Castanopsis indica* | Fagaceae | 2 | 65.0-69.0 | 20.5-29.0 | 0.72 |
| 10 | *Canarium album* | Burseraceae | 1 | 72.0 | 32.4 | 0.61 |
| 11 | *Gironniera subaequalis* | Ulmaceae | 1 | 66.8 | 29.0 | 0.43 |
| 12 | *Cinnamomum bejolghota* | Lauraceae | 8(1) | 30.6-88.0 | 24.8-33.4 | 0.50 |
| 13 | *Litsea pierrei* | Lauraceae | 1(1) | 25.5 | 23.2 | 0.54 |
| 14 | *Croton Argyrata* | Euphorbiaceae | 3(1) | 26.4-47.0 | 20.5-27.5 | 0.50 |
| 15 | *Craibiodendron scleranthum* | Ericaceae | 1 | 80.0 | 24.0 | 0.78 |
| 16 | *Dipterocarpus alatus* | Dipterocarpaceae | 1 | 24.5 | 22.0 | 0.67 |
| 17 | *Elaeocarpus griffithii* | Elaeocarpaceae | 1(1) | 29.6 | 2.5 | 0.62 |
| 18 | *Euodiabo dinieri* | Rutaceae | 1 | 45.4 | 29.5 | 0.43 |
| 19 | *Endospermum chinense* | Euphorbiaceae | 34(1) | 3.2-105.0 | 5.3 – 36.0 | 0.45 |
| 20 | *Garuga pierrei* | Burseraceae | 40(3) | 4.2-93.0 | 5.9-38.0 | 0.63 |
| 21 | *Lithocarpus ducampii* | Fagaceae | 5(1) | 10.8-84.0 | 16.3-33.5 | 0.89 |
| 22 | *Lithocarpus vestisus* | Fagaceae | 1 | 60.0 | 28.0 | 0.56 |
| 23 | *Machilus odoratissima* | Lauraceae | 9(1) | 30.0-105.0 | 23.5-36.5 | 0.61 |
| 24 | *Madhuca pasquieri* | Sapotaceae | 1 | 18.5 | 22.5 | 0.71 |
| 25 | *Mallotus metcalfianus* | Euphorbiaceae | 1(1) | 14.8 | 10.5 | 0.38 |
| 26 | *Microcos paniculata* | Tiliaceae | 1(1) | 22.6 | 16.8 | 0.54 |
| 27 | *Podocarpus nerrifolius* | Podocarpaceae | 1 | 75.0 | 28.5 | 0.48 |
| 28 | *Michelia mediocris* | Magnoliaceae | 1 | 67.0 | 28.5 | 0.55 |
| 29 | *Nephelium cuspidatum* | Sapindaceae | 4(1) | 16.0-75.0 | 17.7-25.5 | 0.79 |
| 30 | *Nephelium melliferum* | Sapindaceae | 5(1) | 19.1-76.0 | 16.0-30.0 | 0.64 |
| 31 | *Ivingia malayana* | Ixonanthaceae | 1 | 75.0 | 29.0 | 0.76 |
| 32 | *Ormosia balansae* | Fabaceae | 1 | 53.3 | 29.5 | 0.52 |
| 33 | *Parashorea stellate* | Dipterocarpaceae | 30 | 1.9-115.0 | 4.2-46.2 | 0.81 |
| 34 | *Paramichelia braianensis* | Magnoliaceae | 36(3) | 3.1-98.0 | 5.7-39.0 | 0.52 |
| 35 | *Plolyalthia cerasoides* | Annonaceae | 5(2) | 11.5-33.1 | 14.0-25.0 | 0.58 |
| 36 | *Prunus arborea* | Rosaceae | 1 | 58.2 | 29.7 | 0.54 |
| 37 | *Schima superba* | Theaceae | 33(1) | 3.4-84.5 | 3.4-33.0 | 0.7 |
| 38 | *Simplocos cochinchinensis* | Symplocaceae | 3(2) | 11.7-23.1 | 13.7-19.7 | 0.40 |
| 39 | *Sinosideroxlon Bonii.* | Sapotaceae | 30 | 1.8-114.0 | 3.3-33.8 | 0.89 |
| 40 | *Cararium benganense* | Burseraceae | 2(1) | 10.7-60.0 | 17.4-28.3 | 0.56 |
| 41 | *Syzygium cuminii* | Myrtaceae | 6(6) | 12.8-29.3 | 14.4-23.6 | 0.66 |
| 42 | *Syzygium wightianum* | Myrtaceae | 1 | 40.0 | 22.0 | 0.72 |
| 43 | *Syzygium zeylanicum* | Myrtaceae | 5(1) | 29.9-96 | 19.1-33.5 | 0.65 |
| 44 | *Caranium parvum* | Burseraceae | 1 | 62.0 | 28.0 | 0.54 |
| 45 | *Vitex quinata* | Verbenaceae | 1(1) | 46.8 | 22.8 | 0.48 |
|  | Total |  | **300(40)** |  |  |  |

Note: Information on sample trees used to parameterize the allometric models. The numbers in the parenthesis indicate the number of sample trees used for root biomass measurements.
